# Alterations in Immune-Related Defensin Alpha 4 (*DEFA4*) Gene Expression in Health and Disease

**DOI:** 10.1155/2022/9099136

**Published:** 2022-05-28

**Authors:** Fatemah Basingab, Abeer Alsaiary, Shahad Almontashri, Aisha Alrofaidi, Mona Alharbi, Sheren Azhari, Khloud Algothmi, Safiah Alhazmi

**Affiliations:** ^1^Department of Biological Sciences, Faculty of Sciences, King Abdulaziz University, Jeddah, Saudi Arabia; ^2^Immunology Unit, King Fahad for Medical Research, King Abdulaziz University, Jeddah, Saudi Arabia; ^3^Biology Department, College of Sciences, Imam Abdulrahman Bin Faisal University, Dammam, Saudi Arabia

## Abstract

Defensin Alpha 4 (DEFA4) is the fourth member of the Alpha Defensins family known as a part of antimicrobial peptides in the innate immune system. DEFA4 has a strong preference to kill Gram-negative bacteria more than Gram-positive bacteria. In addition, DEFA4 exhibits antiviral activity against human immunodeficiency virus type 1 (HIV-1) *in vitro*. Moreover, DEFA4 can act as an inhibitor of corticosterone production (Corticostatin). On the other hand, alternations in *DEFA4* gene expression have been reported in different disorders such as diseases related to inflammation and immunity dysfunction, brain-related disorders, and various cancers. The up-regulation of *DEFA4* appears to be involved in the malignant transformation or aggressive form of cancer. Interestingly, the modified version of DEFA4 fragment (1–11) was potent and efficient against antibiotic-resistant bacteria. This review provides a general background abSaudi Arabia out *DEFA4* and sheds light on changes in *DEFA4* gene expression in different diseases. The paper also discusses other aspects related to DEFA4 as an antimicrobial and antiviral agent. The research was conducted based on available articles obtained from databases starting from 1988 to the present.

## 1. Introduction

The innate immune response is the first mechanism to protect the host from invading pathogens. This response occurs through the collaboration between many constituent elements of the innate immune system. The innate immune system is composed of anatomical and physical barriers, a variety of cells, cell receptors, soluble mediators, and host defense peptides (HDPs) (e.g., histatins, cathelicidin, defensins, etc.) [1].

Defensins are small (3–5 kDa) cysteine-rich, cationic peptides. Mature defensins contain six cysteine residues (Cys), forming three intramolecular disulfide bonds. Based on the configuration of the disulfide bonds, human defensins are classified into alpha (*α*) and beta (*β*) subfamilies [2]. Human *α*-defensin genes co-locate with *β*-defensin genes on adjacent loci on chromosome 8 (8p22-p23) [3–6].

In *α*-defensins, three disulfide bridges are formed (between Cys1-Cys6, Cys2–Cys4, and Cys3–Cys5 pairings) [7]. In humans, the *α*-defensin family consists of six members (DEFA1–6). Members 1–4 were initially identified in neutrophils. Thus, DEFA1–4 were called Human Neutrophil Peptides (HNPs) (HNP-1, HNP-2, HNP-3, and HNP-4) [8, 9]. The other two members were found in the Paneth cells of the intestinal tract. These intestinal defensins are known as Human Defensin (HD5 and HD6) [5]. In this review, the DEFA abbreviation will be used instead of (HNP or HD) to indicate these peptides. DEFA1–4 are abundantly expressed and stored in azurophil granules of neutrophils, where they comprise up to 30% of the protein content in these granules. Furthermore, DEFA1–4 can be expressed by various cell types such as lymphocytes, monocytes, natural killer (NK) cells, and mucosal surface epithelium [10–12].

Human *α*-defensins exert broad-spectrum activities against enveloped viruses and different types of cellular pathogens [13–16]. Additionally, *α*-defensins can act as immunomodulatory agents exhibiting both anti-inflammatory activity and pro-inflammatory activity [17–19]. Human *α*-defensins are chemoattractant for multiple different immune cell types such as immature dendritic cells (DCs), macrophages, mast cells. In addition, *α*-defensins can induce cytokine and chemokine production [11, 17, 20]. The *α*-defensins are released from dead neutrophils by apoptosis or necrosis leading to repressed inflammatory cytokines and nitric oxide release from macrophages which ultimately lead to limiting the pro-inflammatory response [18]. Moreover, *α*-defensins roles are not limited to fighting against pathogens or modulating the immune response. The *α*-defensins have therein been reported to be involved in various non-immune physiological processes such as wound healing and promoting collagen expression [21–23], activation of the Nuclear Factor Kappa B (NF-*κ*B) pathway [20], anti-angiogenic, anti-tumor as well as activation of cancerous cell proliferation [24–28], anti-Adrenocorticotropic hormone (ACTH) activity and inhibiting glucocorticoid hormone secretion [9]. The ability of *α*-defensins to perform various functions has been attributed to the amphiphilic–cationic organization of these peptides. The cationic nature enables these peptides to selectively bind and disrupt the negatively charged microbial membranes. Furthermore, the cationic nature of *α*-defensins may facilitate interactions with different host receptors via electrostatic forces [5].

**Figure 1:**
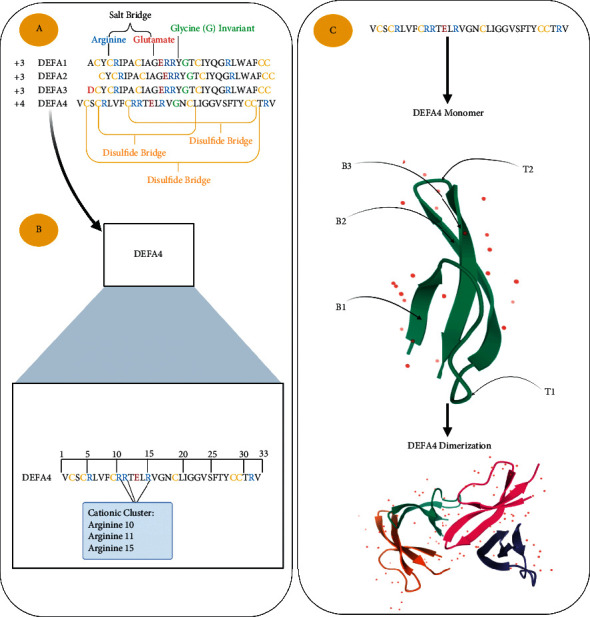
This figure shows the structure-related DEFA4 properties. Created with BioRender.com. (a) Alignments of the amino acid sequences that show conserved residues: 6 Cysteine residues (C) are shown in yellow; Glycine (G) is colored in green, Arginine (R) is located after the second Cysteine, Glutamic acid (E). Positively-charged residues are colored in blue, negatively-charged residues are colored in red. Relative to DEFA1–3 (+3), DEFA4 is the higher positive charge of (+4). This figure also shows the bonds that stabilize the *α*-defensins tertiary structure, 3 disulfide bridges between 6 conserved (C) and one salt bridge formed by the side chains of R and (E). (b) Three clustered cationic residues of Arginine (Arg10, Arg11, Arg15). (c) DEFA4 consists of three beta-sheets (B) arranged into an antiparallel structure. B1and B2 are connected by the long loop (T1), and beta-hairpin (T2) is formed by B2 and B3. DEFA4 forms homodimers [46].

At the peptide sequence level, DEFA1, 2, and 3 are almost identical; DEFA1 and DEFA3 therein differ only at the *N*-terminal residue (alanine in DEFA1 and aspartic acid in DEFA3). Interestingly, DEFA2 is not an individual gene product and it can be encoded by both *DEFA1* and *DEFA3* genes redundantly. DEFA2 can therein be generated through proteolytic removal of the first *N*-terminal amino residue of DEFA1 or DEFA3 [29]. On the other hand, DEFA4 is different from DEFA1–3 at the amino acids sequence level. DEFA4 shares only 11 identical residues with DEFA1, 2, 3. Nine of these eleven shared residues are therein structurally conserved in mammalian *α*-defensins (six Cys residues) (Arginine5 (Arg)—Glutamic acid13 (Glu) ion pair) and (Glycine 17 (Gly)) (Figure 1(a)) [13, 30]. Despite these differences in sequence identity between DEFA1, 2, 3 and DEFA4, the tertiary structures of DEFA4 and DEFA1, 2, 3 are similar [16, 30].

Interestingly, the proteolytic digestion process of *α*-defensins may naturally occur in the human body generating small *α*-defensins-derived peptides [31]. These small *α*-defensins-derived peptides can exhibit distinct biological properties. Ehmann et al., in 2019 observed that full-length DEFA5 can be degraded by human duodenal fluid into fragments and showed various antimicrobial activities as well as modulation of the microbiota composition with maintaining the diversity [31]. DEFA5 (1–9) was therein capable of increasing the *Akkermansia* sp amount in the small intestine microbiota of treated mice for 2 weeks [31]. Moreover, in 2020, DEFA5 (1–9) was reported as an active anti-viral against primary and multi-resistant isolates of Human Cytomegalovirus (HCMV). DEFA5 (1–9) can prevent viral entry by inhibiting HCMV attachment to host cells [32]. In addition, Ehmann et al., in 2020 examined the other *α*-defensins member (DEFA4) and they reported the modified version of DEFA4 (1–11) was highly effective against multidrug-resistant bacteria more than DEFA4 full-length peptide [33].

DEFA1, 2, 3 account for up to 30% of the total protein content in azurophil granules of human neutrophils. In contrast, DEFA4 is considered much less abundant. DEFA4 constitutes only 1% to 2% of the total defensins found in neutrophils [34]. Little is known about DEFA4 compared to DEFA1, 2, 3 which have been extensively studied [30, 35]. To our knowledge, no prior reviews have focused on human *DEFA4.* Therefore, this review attempts to provide sufficient knowledge about human *DEFA4* by discussing the most prominent reports about *DEFA4* milestone discoveries from 1988 to date, along with the observed changes in *DEFA4* expression in different diseases.

## 2. Study Design

Information is obtained from electronic databases including Medline, PubMed, ScienceDirect, Microsoft Academic. The search is limited to publications in the English language from 1988 until 2022. The search is applied based on keywords that included the gene and protein scientific names and symbols. The scientific terms used are Defensin Alpha4, Neutrophil Defensin4, Human neutrophil peptide-4, Human Neutrophil *α*-Defensin 4, Corticostatin, whereas the scientific symbols are: *DEFA4*, *DEF4*, HP4; HP-4; HNP-4.

## 3. Human Defensin Alpha 4 (*DEFA4*)

### 3.1. DEFA4 Milestone Discoveries

**Figure 2:**
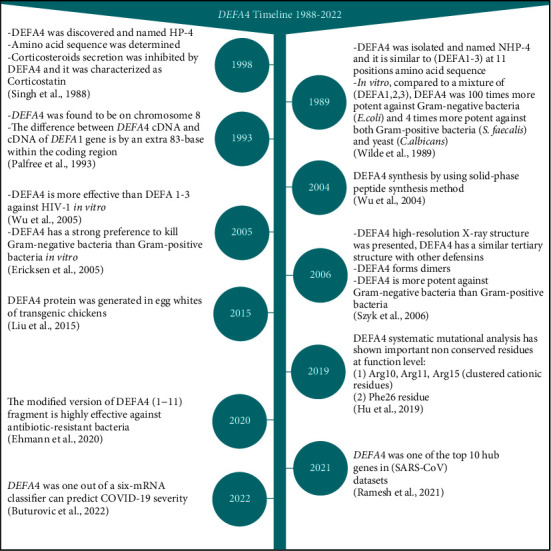
Milestone reports that have contributed to describing *DEFA4* properties over the years. Created with BioRender.com.

Human defensin alpha 4 was discovered and denoted for the first time as HP-4 in 1988 by Singh et al. This discovery came when they were trying to extract human neutrophil peptides that can exhibit anti-ACTH activity (corticostatic activity). They found that at nanomolar concentrations, the novel peptide has exhibited corticostatic activity on the rat adrenal cell suspension *in vitro*. Thus, due to this corticostatic activity, DEFA4 was also described as Corticostatin [9]. Later in 1989, Wilde et al. also announced the discovery of the same novel peptide that had been previously discovered in 1989 by Singh et al., and it was called Human Neutrophil Peptide-4 (HNP-4). Moreover, they observed the preferential ability of DEFA4 to kill Gram-negative bacteria more than Gram-positive bacteria [13]. In 1993, Palfree et al. found that *DEFA4* was located on chromosome 8. In addition, at the sequence level, the complementary DNA (cDNA) of the *DEFA4* gene was similar in approximately 72% of the nucleotide sequence identity of the *DEFA1* gene. Nevertheless, the cDNA of the *DEFA4* gene was different from the cDNA of the *DEFA1* gene by an extra 83-base segment that appeared to be the result of a recent duplication within the coding region [4]. Due to DEFA4 scarcity in natural sources, previous studies have attempted to produce DEFA4 peptides [35, 36]. In 2004, DEFA4 was synthesized using the solid-phase peptide synthesis method [35]. In 2005, DEFA4 anti-microbial ability was assayed compared to other *α*-defensins members, and the potency of DEFA4 against Gram-negative bacteria was confirmed [14]. Moreover, DEFA4 was investigated as an antiviral agent *in vitro*, and DEFA4 was observed to act effectively against human immunodeficiency virus type 1 (HIV-1) (detailed in DEFA4 functions) [15]. Later in 2006, the high-resolution X-ray structure of DEFA4 was presented by Szyk, and the similarity in the tertiary structure of DEFA4 with other members of *α*-defensins and *β*-defensin was demonstrated (detailed in DEFA4 protein structure). Additionally, DEFA4 anti-microbial activity has been assessed, confirming the strong ability of DEFA4 against Gram-negative [16]. In the context of production, antimicrobial peptides are an alternative to antibiotics to overcome the drug resistance-bacteria crisis. In 2015, transgenic chickens capable of expressing the DEFA4 protein in egg whites were generated [36]. In 2019, systematic mutational analysis was conducted to elucidate the molecular determinants underlying DEFA4 functions. All DEFA4 residues were individually mutated to alanine (Ala) except for the nine conserved residues (6xCys, 1xArg, 1xGlu, and 1xGly). They have pointed to the important functional residues in DEFA4 (detailed in DEFA4 protein structure) [30]. In 2020 Ehmann et al. reported that the modified version DEFA4 (1–11) was highly potent and efficient against antibiotic-resistant bacteria, suggesting DEFA4 (1–11) can be used as a cost-effective therapeutic candidate [33]. *DEFA4* has been identified as one of the top hub genes represented as nodes with a high degree of interaction in the network of differentially expressed genes in acute respiratory syndrome coronavirus (SARS-CoV) datasets. This suggests that *DEFA4* may play an important role in SARS-CoV infection pathology [37, 38]. Hub genes produce proteins that can interact with a large number of other proteins (partners) [39]. The hub genes are considered functionally significant in the pathogenesis and progression mechanism of many diseases. Moreover, the hub genes may represent candidate biomarkers for diagnosis as well as be targeted in the therapeutic procedure. For example, *DEFA4* was one of 6 genes constructed as a classifier that can predict the severity of different viral infections including the SARS-CoV 2 virus (COVID-19) [40]. All studies are summarized in Figure 2.

### 3.2. DEFA4 Gene Expression

**Figure 3:**
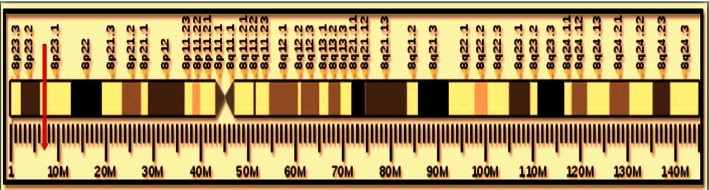
*DEFA4* cytogenetic location in the human genome (8p23.1.), *DEFA4* gene is therein located on the short arm (p) of chromosome 8 at position 2, band 3, sub-band 1.

Defensin Alpha4 gene (*DEFA4*) is a protein-coding gene that results in the production of Defensin Alpha4 peptides (DEFA4), also known as Human Neutrophil Peptide 4 (HP-4, HNP-4, HP4), Neutrophil defensin4, and Corticostatin. *DEFA4* gene is located on chromosome 8, specifically 8p23.1 (Figure 3) [41]. *DEFA4* gene consists of three exons, the first exon encoding a 5′ untranslated region. Therefore, only two exons are present in the final version of mature RNA (mRNA) as a protein-coding sequence [42]. At the transcription level, only one transcript is produced that is translated into the mature peptide containing only 33 residues [43]. In normal human tissues, *DEFA4* is expressed in various tissues, but the highest *DEFA4* expression rate was found in bone marrow and whole blood [41, 44].

### 3.3. DEFA4 Protein Structure

During the translation process, the mRNA of *DEFA4* is translated as a 97-residue precursor. Cleavage of the DEFA4 precursor occurs at several sites resulting in mature peptides containing only 33 residues. DEFA4 mature sequence is: HN2-Val-Cys-Ser-Cys-Arg-Leu-Val-Phe-Cys-Arg-Arg-Thr-Glu-Leu-Arg-Val-Gly-Asn-Cys-Leu-Ile-Gly-Gly-Val-Ser-Phe-Thr-Tyr-Cys-Cys-Thr-Arg-Val-COOH [13, 45]. Out of the 33 residues, nine conserved residues are present on all defensins, six Cys residues,1 Arg, 1 Glu, and 1 Gly residue. Moreover, the six Cys residues form intramolecular disulfide bonds. Arg (5) and Glu (13) form the invariant salt bridge, which has a role in cellular stability and biosynthesis (Figure 1(a)) [16]. Gly (17) is important for correct folding [30]. The folding of DEFA4 monomers is arranged into three antiparallel beta-sheets (B) (Figure 1(c)) [16]. The conserved residues are the most structurally conserved and rigid residues present in the peptide sequence and play roles in folding, proteolytic stabilizing, and chemotactic activity. Any changes or substitutions of these residues are structurally harmful [16]. Arg10, 11, 15 are important cationic residues that have a role in the elimination of bacteria, while Phenylalanine (phe26) is the key residue for most of the DEFA4 functions [30].

### 3.4. DEFA4 Functions

#### 3.4.1. DEFA4 as Inhibitor of Corticosterone Production (Corticostatin)

ACTH stimulates adrenocortical steroidogenesis to produce corticosterone (named Cortisol in humans) [47]. However, this stimulation action can be interfered with and inhibited by corticostatic activity (anti-ACTH). In 1988, when DEFA4 was discovered, DEFA4 ability to exert corticostatic activity was examined on rat adrenal cell suspensions *in vitro*. It was reported that, at nanomolar concentrations, DEFA4 can interfere with ACTH and inhibit corticosterone production. As a result of this DEFA4 functional property, DEFA4 was described and termed Corticostatin [9].

#### 3.4.2. DEFA4 Anti-Microbial Activity

One of the most known features of DEFA4 is the preferential ability to kill Gram-negative bacteria more than Gram-positive bacteria. In 1989 Wilde et al. reported DEFA4 was 100 times more efficient against Gram-negative bacteria *Escherichia coli* (*E. coli*) and four times more against both Gram-positive bacteria *Streptococcus faecalis* (*S. faecalis*) and yeast *Candida albicans* (*C*. *albicans*) in comparison to DEFA1, 2, 3 mixture [13]. This finding was consistent with Ericksen's results in 2005, when DEFA4 showed a stronger preference to kill Gram-negative bacteria *E*. *coli* and *Enterobacter aerogenes* (*E*. *aerogenes*) than other members (DEFA1, 2, 3) whereas, DEFA5 was comparable to DEFA4. Conversely, DEFA4 was less active against Gram-positive bacteria *Staphylococcus aureus* (*S*. *aureus*) and *Bacillus cereus* (*B*. *cereus*) [14]. In another study in 2006, DEFA4 was reported to be more potent against the Gram-negative bacteria *E*. *coli* than Gram-positive bacteria *S*. *aureus*, and yeast *C*. *albicans* [16]. This preferential ability of DEFA4 to kill Gram-negative bacteria is associated with its cationic properties and distinctive distribution of positively charged amino acids in the DEFA4 structure. DEFA4 has the extra positive charge (+4) compared to DEFA1, 2, 3 (+3). Moreover, DEFA4 has a positive cluster that is composed of three clustered cationic amino acids (Arg10, Arg11, and Arg15). The cationic cluster plays an important role in facilitating high-affinity interactions with negatively charged components lipopolysaccharide (LPS) in the outer membrane of Gram-negative bacteria (Figure 1(b)) [30].

On the other hand, the small *α*-defensins-derived fragments display distinctive biological properties that exceed the full-length peptide [31, 33]. Recently, a DEFA4-derived fragment (1–11) was generated by proteolytic digestion to investigate the antimicrobial activity of this fragment *in vitro*. Moreover, in aiming to enhance the stability against proteolytic digestion, a DEFA4 (1–11) modified version was generated by acetylation of the *N*-terminus, amidation of the *C*-terminus, and exchanging the *L*-amino acids with *D*-amino acids. Results indicated that the modified version DEFA4 (1–11) showed high potency as well as high efficacy in comparison with the full length of DEFA4 and the non-modified version DEFA4 (1–11) [33]. Interestingly, the modified version DEFA4 (1–11) was highly effective against *Pseudomonas aeruginosa*, *Klebsiella pneumoniae* and *Acinetobacter baumannii* that were considered the top pathogenic bacteria listed as multidrug-resistant bacteria according to Centers for Disease Control and Prevention (CDC) and the World Health Organisation (WHO) global priority pathogens list [33, 48, 49]. Thus, modified version DEFA4 (1–11) may serve as a promising alternative therapy to overcome the antibiotic-resistant bacteria crisis.

#### 3.4.3. DEFA4 Antiviral Activity

The HIV-1 infection cycle begins when specific viral surface protein (viral envelope glycoprotein 120 (gp120) recognizes and binds to specific *T* cell receptors (the cluster of differentiation four receptor (CD4 receptor)) [50]. The *α*-defensins can block the initial phase of the HIV infectious cycle by acting as a lectin ligand of CD4 and HIV-1 gp120, preventing CD4-gp120 interaction. Besides, *α*-defensins can dramatically down-regulate the expression of CD4 receptors that are considered critical receptors in *T*-cell activation [51]. Wu et al. examined the antiviral ability of DEFA4 along with DEFA1, 2, 3 against both X4 and R5 strains of HIV-1 *in vitro*. DEFA4 was observed to be more effective than DEFA1, 2, 3 in protecting peripheral blood mononuclear cells (PBMCs) from infection by both strains of HIV-1 [15], although DEFA4 has a significantly weaker capacity in acting as a lectin ligand to gp120 and CD4. Thus, HIV-1 inhibition by DEFA4 is attributed to a lectin-independent property, but the exact mechanism underlying this effective inhibition is still unclear [15, 52].

As mentioned in the introduction, DEFA5-derived fragments served as promising therapeutic agents [31–33]. DEFA 5 (1–9) and DEFA5 (7–32) showed more anti-viral activity against HCMV than DEFA4 (1–11). Notably, DEFA5 (1–9) showed antiviral activity against HCMV primary and multiresistant HCMV strains with lower cytotoxicity. DEFA5 (1–9) can prevent HCMV infection through inhibition of HCMV attachment to various human cells. At the structure level, four amino acids showed functional importance for DEFA 5 (1–9) antiviral activity against HCMV, Cys3, and Cys5 that contribute to disulfide bridges formation as well as Arg6 and Arg9 which are important for membrane interactions [32].

### 3.5. DEFA4 Gene in Infectious and Inflammatory Diseases

#### 3.5.1. Liver-Related Diseases

The primary function of *DEFA4* is to eliminate pathogenic microorganisms such as viruses, bacteria, fungi, and yeast. Through the years, a strong connection between infectious diseases and *DEFA4* has been established (Table 1 summarizes the studies that have investigated changes in *DEFA4* gene expression related to infectious and inflammatory diseases). Several reports indicated the changes in the *DEFA4* gene expression level are associated with liver pathology such as the different kinds of hepatitis infection including hepatitis B virus (HBV), hepatitis C virus (HCV), and hepatitis E virus (HEV). A study was published by Zhou et al. to identify the pathogenesis of hepatitis B–related acute chronic liver failure (HBV-ACLF). This study concluded that *DEFA4* is among one of the genes that could serve as potential biomarkers for HBV-ACLF detection [53]. Severe viral infection and increased inflammation could be significant risk factors for HBV-ACLF development. Thus, the high levels of *DEFA4* may be attributed to its function as a host defense peptide in the inflammatory response [53]. Another study conducted by Qiu et al. aimed to analyze gene expression profiles of PBMCs from patients that failed to trigger effective immune response after HBV vaccination. *DEFA4* was reported to be up-regulated in the non-responders [54]. A high level of *DEFA4* can be produced by cytokines or the activation of toll-like receptors (TLRs) that can modulate adaptive immune response [54]. TLRs are pattern-recognition receptors that play a key role in the innate immune response. TLRs act as initiators of the innate immune response by enabling the host to identify pathogen-associated molecular patterns (PAMP). In general, defensins can prevent viral infection by directly acting on the virion or by affecting the target cell and hence interfering with viral infection indirectly [55].

Furthermore, the peripheral blood samples from patients with HCV submitted to liver transplant have been examined after seven days of the transplantation. The result indicated that *DEFA4* was up-regulated in recipients [56]. In 2020 a study was conducted by Ramadasi and Arankalle among pregnant women with HEV to investigate the possible mechanisms that lead to the presence of this infection. The study examined women at the 2nd and 3rd trimesters, and *DEFA4* was observed at a high level among these pregnant patients [57]. This *DEFA4* up-regulation only in pregnant women may be attributed to gene expression changes that naturally occur during pregnancy. According to Knight et al., *DEFA4* was one of the genes that showed an increased expression throughout a healthy full-term pregnancy [58].

#### 3.5.2. Respiratory-Related Diseases and Periodontitis

Additionally, numerous studies have revealed up-regulation of *DEFA4* in various respiratory-related diseases. Interestingly, a study conducted by Overmyer et al. regarding COVID-19 on 102 COVID-19 patients sought to predict its severity and possible pathophysiology. The elevated level of *DEFA4* expression was correlated with the status and severity of symptoms in COVID-19 patients [59]. Up-regulation of *DEFA4* and neutrophil-related genes in patients with COVID-19 indicated an increased number of neutrophils and degranulation [59]. Consequently, neutrophils release the inflammatory mediators and contribute to cytokines storm [60]. In addition, neutrophils can release neutrophil extracellular traps (NETs) that can be defined as extracellular webs comprised of granule proteins, oxidant enzymes, and chromatin. These NETs may increase inflammation and microvascular thrombosis leading to organ damage [61, 62]. Moreover, it was reported that elevated levels of both Interleukin 6 (IL-6) and *α*-defensins were associated with the acceleration of clot formation and disease severity [62]. Furthermore, up-regulation of *DEFA4* and neutrophil-related genes may reflect emergency myelopoiesis in fatal COVID-19 [63]. On the other hand, dexamethasone treatment was recommended for only those COVID-19 patients undertaking respiratory support; wherein, dexamethasone treatment was reported to reduce the deaths in ventilated patients and patients receiving oxygen therapy [64]. Dexamethasone is the synthetic version of cortisol hormone that exhibits the same anti-inflammatory effects of cortisol. Cortisol is naturally produced in the human body and plays a critical role in inhibiting inflammation through acting as a transcriptional factor and regulating the gene expression of inflammatory-related genes [65]. *DEFA4* exhibited corticostatic effects at nanomolar concentrations [9]. Thus, another possible effect is that *DEFA4* may indirectly contribute to exacerbating the inflammatory state through interfering with Hypothalamic-Pituitary-Adrenal axis (HPA axis) activity and acting as anti-ACTH leading to inhibiting the production of cortisol. Consequently, the produced cortisol in COVID-19 patients may not meet the required bodily need to inhibit inflammation [66].

Obviously, *DEFA4* has a significant influence on respiratory-related diseases such as asthma and idiopathic pulmonary fibrosis (IPF). In 2017, a study was conducted by Bigler et al. aiming to identify the differences between non-asthma and asthma patients through a gene expression analysis approach. It was found that *DEFA4* was significantly associated with the severity of asthma. Chemotaxis, migration, and myeloid cell trafficking were found to be enhanced in patients with severe asthma, whereas B-lymphocyte development, hematopoietic progenitor cells, and lymphoid organ hypoplasia were found to be at a low level [67]. Moreover, another study published in 2018 targeted children with asthma to characterize the possible phenotypes of this disease. Among 19 up-regulated genes, *DEFA4* was remarkably present in the neutrophil-predominant asthma phenotype compared to other asthma subtypes [68]. Childhood asthma phenotypes can be distinguished by either eosinophil-predominant or neutrophil-predominant inflammatory features. The great majority of differentially expressed genes in neutrophil-predominant asthma were related to corticosteroid response or anti-corticosteroid response [68].

Furthermore, *DFEA4* was among 13 expressed genes that can distinguish between mild and severe IPF cases [69]. Another study regarding IPF demonstrated *DEFA4* as one of the highly expressed genes in IPF patients [70]. One of the possible links between up-regulated *DEFA4* and the severe forms of respiratory-related diseases is that up-regulation of *DEFA4* may reflect an excess of NETs formation. Excess NETs can spread in the pulmonary alveoli leading to lung damage. Along with this, these NETs can contribute to endothelial and epithelial cell death [71, 72].

In oral-related diseases, *DEFA4* was observed at low levels among periodontitis patients compared to the healthy subjects, whereas the low expression level of the *DEFA4* gene could serve as a potential biomarker to detect periodontal conditions [73].

#### 3.5.3. Autoimmune Diseases

Moreover, *DEFA4* has an effective role in autoimmune diseases such as systemic lupus erythematosus (SLE). A study conducted by Villanueva et al. proposed that the low-density granulocytes (LDGs) have a potential role in the appearance of lupus erythematosus. The results showed a high level of *DEFA4* in the LDGs. LDGs are distinct immature neutrophils subsets found in SLE patients with the ability to enhance the formation of Neutrophils extracellular traps (NETs) and up-regulate the expression of enzymes and neutrophil proteins implicated in the NETs formation. NETs are an important phenomenon in autoantigen modification and exposure to the immune system, as well as in the induction of tissue damage. NETs formation plays an important role in the autoimmune disease development and organ damage observed in chronic inflammatory disorders [74]. The results also showed *DEFA4* is related to granulopoiesis genes that were found to be up-regulated in the LDGs in SLE patients [74]. Moreover, another study was applied in monozygotic twins discordant for multiple systemic autoimmune diseases (SAID), as well as compared to unrelated, matched controls. A high level of *DEFA4* was observed in patients suffering from SAID [75].

### 3.6. Nervous System-Related Diseases and DEFA4

#### 3.6.1. Psychiatric and Neurodegenerative Diseases

Increasing evidence has indicated that an inflammatory component is involved in psychiatric disorders and different brain-related diseases. Many studies have investigated the levels of inflammatory mediators in patients with psychiatric disorders [76–79]. For example, plasma samples were used to measure protein levels of *α*-defensins from 21 monozygotic twins discordant for schizophrenia (SZ) and 8 unaffected twin pairs as controls. Elevated *α*-defensin levels have been identified in both affected and unaffected monozygotic twins discordant for SZ compared to healthy twin pairs. Notably, *α*-defensin levels in the unaffected discordant twins were at an intermediate level (more than healthy twin pairs but less than their schizophrenic twins). Thus, it was suggested that increased *α*-defensin levels might serve as an indicator of susceptibility to develop SZ [76]. On the other hand, *DEFA4* showed increased levels in patients with different psychiatric disorders (Table 2). For instance, Gardiner et al. in 2013 found that *DEFA4* was one of the 59 up-regulated genes in PBMCs samples in SZ patients and schizoaffective disorder patients compared with controls [77]. Furthermore, DEFA4 was observed as one of the eight proteins that showed increased levels in saliva samples of SZ and bipolar disorder (BD) patients versus the control group [78]. Recently, *DEFA4* was observed to be up-regulated in Japanese women with post-traumatic stress disorder (PTSD) who have elevated levels of IL-6 compared with those who have normal levels of IL-6, suggesting that *DEFA4* up-regulation appeared to be correlated with high levels of IL-6 [80].

Furthermore, altered *DEFA4* gene expression was found in different neurodegenerative diseases such as Alzheimer's disease (AD) and Parkinson's disease (PD) (Table 2). Recently, Cohen et al. reported that *DEFA4* was one of the 50 genes that showed significant differences in gene expression in blood samples of AD and mild cognitive impairment (MCI) versus healthy individuals. *DEFA4* was therein observed to be up-regulated in both AD and MCI compared to individuals with the normal cognitive ability [81]. On the other side, *DEFA4* was found as one of the 13 common genes that showed a significant differential gene expression in blood samples of both genetic-associated PD and idiopathic PD. Wherein *DEFA4* was up-regulated in both genetic-associated PD compared with asymptomatic carriers, as well as between idiopathic PD compared with controls [82]. On the contrary, in another study, *DEFA4* was reported as one of the top 20 genes with differential gene expressions in blood samples of PD patients. *DEFA4* was therein down-regulated in PD patients compared to healthy controls [83].

It is unknown why *DEFA4* showed differential gene expression in patients with various diseases related to the brain. This up-regulation of *DEFA4* may be a consequence of using antipsychotics. According to Benedicto et al. (2019), *DEFA4* up-regulation was observed after three months of treatment with antipsychotic medications [84]. On the other hand, *DEFA4* up-regulation could reflect the inflammatory state underlying brain-related disorders, and not a result of using antipsychotics. This notion is supported by the aforementioned fact that elevated *α*-defensin levels were identified in unaffected and untreated monozygotic twins discordant for SZ compared to healthy unaffected twins [76].

From our knowledge of *DEFA4*, there are possible explanations. First, changes in *DEFA4* gene expression may not be related to brain pathogenesis and could be a consequence of an inflammatory state in the body. As mentioned above in Hori et al, *DEFA4* was up-regulated in PTSD patients who have high levels of IL-6 compared to other patients with normal levels of IL-6 [80]. This positive correlation between *α*-defensins and IL-6 was also recorded in other diseases such as COVID-19 [62]. This may indicate that the up-regulation of *DEFA4* could be a consequence of increased IL-6 levels. It was therein reported that IL-6 can activate neutrophils and stimulate the release of *α*-defensins from human neutrophils *in vitro* [62]. High-level IL-6 was observed in blood samples of patients with different brain-related disorders [85–87]. Although the blood state does not necessarily reflect the brain state, IL-6 can cross the blood-brain barrier (BBB) [88]. The presence of IL-6 at excessive levels in the central nervous system (CNS) may contribute to the neuroinflammation that ultimately leads to brain-related disorders [86]. On other hand, hypercortisolism was reported to be associated with changes in structure and function of the brain leading to impaired brain ability [89]. Thus the second possibility is that the up-regulation of *DEFA4* as corticostatin may reflect the immunomodulatory role for the HPA axis in conditions of hypercortisolism [9, 90].

#### 3.6.2. Multiple Sclerosis

Patients with Multiple sclerosis (MS) go through two main phases of remission and relapse. The relapsing phase is when the neurologic symptoms increase and appear as attacks, followed by the remitting phase when the patients recover partially or completely [91]. *DEFA4* was found to be up-regulated in blood samples of MS females in remission status [92]. De Andres et al., 2018 reported that *DEFA4* was one of seven highly up-regulated genes in CD4+ *T* lymphocytes of MS patients after 3–6 days treated by IVMP. Intravenous methylprednisolone (IVMP) is one of the known treatments for acute relapses of MS [93]. On the other hand, gene expression analysis was applied on the post-mortem of normal-appearing white matter (NAWM) samples from MS patients and compared with controls. Different modules have been identified; one of these modules showed a strong positive correlation to both MS severity and HPA axis activity. *DEFA4* was identified as one of the top ten genes strongly connected to this module [94]. As mentioned before, cortisol is produced by the HPA axis and classified as the primary corticosteroid in humans [47, 65]. Corticosteroids are commonly used as a treatment for MS patients as anti-inflammatory drugs [91]. DEFA4 as a corticostatin substance may be related to HPA axis activity and cortisol production which may affect disease progression. Future research is required to identify how *DEFA4* is involved in pathways related to MS disorder (Table 2).

### 3.7. Cancer

In an attempt to detect cancer signature genes, bioinformatics analysis was applied on approximately 1500 microarray gene expression profiles from 26 cancer studies across 21 human cancer types. According to this analysis, *DEFA4* has been concluded as one of 48 genes identified as common cancer signature genes [95]. Head and neck cancers are the most common cancer types that appear to be associated with changes in *DEFA4* gene expression levels [96, 97]. Wenghoefer et al., reported an increased gene expression of *DEFA4* (179.2 fold) in oral leukoplakia biopsies compared with healthy gingiva [97]. Leukoplakia can be described as a premalignant lesion in the oral cavity that occurs in an intermediate stage before malignant [98]. Thus, it was suggested that *DEFA4* up-regulation may play a role in malignant transformation [97]. Later in 2012, Winter et al. provided further evidence supporting previous observations regarding *DEFA4* up-regulation and its potential role in malignant transformation. Elevated level *DEFA4* gene expression was observed in all tested subsets of salivary gland tumors. Conversely, a significant decrease in *DEFA4* gene expression was reported in benign mixed tumors (pleomorphic adenomas) [96]. Altered *DEFA4* gene expression has been observed in bone marrow-related cancers. Interferon-alpha2 is considered a therapeutic agent for Philadelphia-negative chronic myeloproliferative neoplasms (MPNs). In the context of using interferon-alpha2, gene expression of interferon-associated genes was analyzed using blood samples from patients with different phenotypes of MPNs: Essential thrombocythemia (ET), Polycythemia vera (PV), Primary myelofibrosis (PMF). *DEFA4* was reported as one of the top ten up-regulated genes in patients with PMF compared with controls [99]. Furthermore, *DEFA4* up-regulation was one of the five distinct gene signatures that can be used as an indicator of disease transition from ET and PV to the aggressive phenotype or transform towards myelofibrosis [100] (Table 3).

The over-activation of epidermal growth factor receptor (EGFR) pathways plays a critical role in the progression of cancer cell proliferation and promotes tumorigenesis. In 2016 Hoppe et al., found DEFA4 (113%) stimulated proliferation on the (BHY) cell line. EGFR signaling pathways can be activated by DEFA4, promoting cyclin D1 activation, which ultimately leads to increased cell proliferation and tumor growth. DEFA4 ability to activate EGFR signaling pathways may be attributed to DEFA4 and epidermal growth factor (EGF) structural similarity, suggesting that DEFA4 may serve as a ligand for EGFR and may be involved in tumorigenesis [24].

## 4. Conclusion and Further Directions

This review set out to better understand the fourth member of the human *α*-defensin family by reviewing the available information about DEFA4. Based on the previous literature, the current review pointed to the potential role of *DEFA4* in two areas: First, the probable role of *DEFA4* in carcinogenesis that could occur through affecting the EGFR pathway. Future research is required to confirm whether *DEFA4* could be used as an early indicator of malignant transformation risk. Second, *DEFA4* may contribute to the modulation process of HPA activity. Further research is needed to assess the association between *DEFA4* expression, IL-6, HPA axis hormones in patients with brain-related disorders as well as in the other diseases treated by corticosteroids. Third, further research is recommended to discover the full therapeutic potential of DEFA4 fragments generated by proteolytic digestion.

## 5. Limitations of This Study

A few limitations in the review should be considered: Many studies reported changes in *DEFA4* gene expression in different diseases. However, there was a lack of detail about DEFA4-related molecular pathways. Furthermore, regarding DEFA4 function in immunity, we cannot completely rule out the impact of infection or the response to drugs that might be responsible for changes in *DEFA4* gene expression. Despite these limitations, this review has provided a general background on *DEFA4* and significant observations about *DEFA4*-related diseases that may open the door for further research.

## Figures and Tables

**Table 1:** List of studies that indicated an alteration in *DEFA4* gene expression in respiratory-related diseases, periodontitis, liver-related diseases, autoimmune diseases.

References	Diseases	Study aim	Research contribution
[53]	Hepatitis B virus (HBV)	To identify the possible causes and pathogenesis of hepatitis B-related acute chronic liver failure (HBV-ACLF); the analysis of the transcriptome of PBMCs has been applied.	*DEFA4* gene was indicated as one of the genes that could serve as a potential biomarker for HBV-ACLF detection.

[54]	Hepatitis B virus (HBV)	Analysis of the transcriptome of PBMCs to recognize the gene expression pathways that failed to trigger effective immune response after a hepatitis B vaccination.	*DEFA4* gene was up-regulated in the non-responders.

[56]	Hepatitis C virus (HCV)	Gene expression profiles were examined on peripheral blood samples from liver transplant recipients with HCV (*n* = 6) before and after the seven-day liver transplantation	*DEFA4* was detected to be one of the 97 up-regulated genes (>2-fold) in liver transplant recipients with HCV seven days after liver transplantation compared with before transplantation

[57]	Hepatitis E virus (HEV)	RNAseq analysis was applied to understand the causes of HEV in: -Pregnant women (PR) at their 2^nd^ and 3^rd^ trimesters at two-phase acute and subclinical phases. -Non-pregnant women (NPR) also with two phases acute and convalescent, all compared with healthy subjects.	The result indicated that there was no presence of *DEFA4* in NPR women at all phases while it was up-regulated in all phases for PR women.

[67]	Asthma	To determine the differences between asthma and non-asthma groups, as well as stratify potential subgroups depending on gene expression of blood samples.	*DEFA4* was one of the genes that showed significantly higher gene expression in patients with severe asthma compared with healthy individuals.

[68]	Asthma	This study aimed to distinguish between different phenotypes of asthma in children through examining gene expression profiles of PBMCs.	(i) *DEFA4* was identified as one of the 19 up-regulated genes in neutrophil-predominant asthma compared with other asthma phenotypes. (ii) 40% of non-responders to corticosteroid treatment were suffering neutrophil-predominant asthma.

[69]	Idiopathic pulmonary fibrosis (IPF)	In this study, gene expression profile analysis of blood samples was performed to identify biomarkers that would enable determining the severity and monitoring the progression of IPF.	*DEFA4* was reported as one of 13 differentially expressed genes that can distinguish between mild and severe IPF cases, wherein *DEFA4* was up-regulated in severe cases.

[70]	Idiopathic pulmonary fibrosis (IPF)	This study aimed to examine the gene expression profiles from blood samples of IPF patients compared to controls at the baseline and longitudinal follow-up after (1, 3, 6) months as well as one year (if alive) to monitor changes in the gene expression over time.	*DEFA4* was one of the top up-regulated genes in IPF subjects versus controls. -Moreover, *DEFA4* was one of the most significant genes that showed higher expression in IPF patients over 12 months.

[59]	Coronavirus disease 2019 (COVID-19)	Multi-omic analysis was conducted on blood samples of COVID-19-positive patients (*n* = 102) and COVID-19-negative patients (*n* = 26) to provide insight into COVID-19 pathophysiology and prediction of its severity.	Increased *DEFA4* expression was reported as one of 219 molecular features that significantly correlated to COVID-19 status and severity.

[73]	Periodontitis	In order to understand the pathogenic process in periodontitis, by RT-qPCR, the expression levels of several AMPs genes were measured in gingival smears from 12 patients with moderate or severe chronic periodontitis and 11 healthy subjects.	(i) *DEFA4* was one of three genes identified to be associated with periodontal health. *DEFA4* was observed to be down-regulated in the periodontitis patients compared with healthy controls. (ii) Moreover, a significant positive correlation was recorded between the expression level of the *DEFA4* gene and the presence of *Porphyromonas gingivalis* and *Parvimonas micra.*

[74]	Systemic lupus erythematos-us (SLE)	This study suggests that the low-density granulocytes (LDGs) have a substantial role in the pathogenesis of lupus erythematosus through the comparison with autologous normal-density neutrophils and control neutrophils.	*DEFA4* gene was among the genes found to be up-regulated in LDGs.

[75]	Systemic autoimmune diseases (SAID)	RNA microarray analysis has been applied to indicate the gene expression in PMBC from 20 monozygotic twin pairs discordant for multiple SAID as well as 40 unrelated control subjects.	*DEFA4* gene was noticed to be up-regulated in subjects with SAID compared to unrelated, matched controls.

**Table 2:** A list of studies reporting *DEFA4* gene expression changes in nervous system-related diseases.

References	Diseases	Study aim	Research contribution
[77]	Schizophrenia (SZ)	Gene expression profiles of PBMCs samples were analyzed from 114 SZ and schizoaffective disorder patients versus 80 healthy controls.	*DEFA4* was found to be one of the 59 genes that were significantly up-regulated in SZ patients and schizoaffective disorder patients versus controls. Moreover, a significant correlation was revealed between *DEFA4* expression and gender; *DEFA4* expression was therein higher in males than females.

[78]	Bipolar disorder (BD) and schizophrenia (SZ)	The proteomics analysis was conducted on saliva samples from 32 SZ patients, 17 patients with BD compared to 31 healthy controls.	DEFA4 has been reported as one of the eight proteins that showed elevated levels in SZ and BD patients compared with healthy subjects.

[80]	Post-traumatic stress disorder (PTSD)	Transcriptome profiles of blood samples from PTSD patients with high IL-6 levels (*n* = 16) and PTSD patients with normal IL-6 levels (*n* = 16) were compared with age-matched normal controls (*n* = 16) (all participants were women).	*-DEFA4* was observed to be up-regulated in PTSD patients who have elevated levels of IL-6 compared with those who have normal levels of IL-6. A significant positive correlation was reported between *DEFA4* expression and serum IL-6 levels.

[81]	Alzheimer's disease (AD) and mild cognitive impairment (MCI)	Gene expression analysis was conducted on blood samples of 200 subjects with a diagnosis of early AD, 400 individuals with MCI, and nearly 200 cognitively normal individuals as the control group.	*DEFA4* was one of 50 genes that have shown the most significant differences between AD and MCI compared to the control group, wherein *DEFA4* was up-regulated in both AD and MCI versus controls.

[82]	Parkinson's disease (PD)	To determine the genes involved in PD pathogenesis (both genetic PD or idiopathic PD) next-generation sequencing (RNA-seq) was conducted on blood samples of: (i) 20 PD patients with the G2019S mutation of the *LRRK2* gene. (ii) 20 asymptomatic carriers of the mutation (iii) 20 subjects with idiopathic PD (iv) 20 controls	*DEFA4* was one of the 13 common genes that were identified to have significant differential gene expression in G2019S-associated PD and idiopathic PD, wherein *DEFA4* was up-regulated in both: (i) (G2019S-associated PD compared with asymptomatic carriers) (ii) (between idiopathic PD compared with controls).

[83]	Parkinson's disease (PD)	To elucidate the molecular basis underlying PD, transcriptomic analyses were performed on blood samples of 72 PD patients compared to 22 healthy controls.	*DEFA4* was reported as one of the top 20 aberrantly expressed genes in PD patients, *DEFA4* down-regulation was found in PD patients compared to healthy controls.

[92]	Multiple sclerosis (MS)	Gene expression analysis was conducted on blood samples from patients with MS in remission, relapsing and healthy controls, aiming to understand the molecular mechanisms underlying MS.	*DEFA4* was one of the genes that up-regulated in females with MS in remission status compared with their controls.

[94]	Multiple sclerosis (MS)	The study aims to analyze the post-mortem of normal-appearing white matter (NAWM) of MS patients to identify the possible gene expression pathways that affect the disease heterogeneity and HPA axis activity.	*DEFA4* was one of the top ten genes that strongly connected to the module with a strong positive correlation to both severity of MS and HPA axis activity

[93]	Multiple sclerosis (MS)	This study aimed to identify the differentially expressed genes in CD4+ *T* lymphocytes of relapsing-remitting MS patients during relapse after 3–6 days of treatment with IVMP *in vivo*.	11 genes were found to be the most significantly differentially expressed in CD4+ *T* lymphocytes of patients after treatment with IVMP. *DEFA4* was one of seven genes that were highly up-regulated.

**Table 3:** List of studies that observed changes in DEFA4 gene expression in different cancer types.

References	Diseases	Study aim	Research contribution
[95]	Different types of human cancer	Bioinformatics analysis has been applied on ∼1500 microarray gene expression profiles representing 21 main human cancer types.	DEFA4 was reported as one of the 46 genes identified as a common cancer signature.

[97]	Oral leukoplakia	The transcript levels of oncogenes, and genes involved in inflammation, and antimicrobial activity were measured in leukoplakia biopsies (*n* = 20) compared with healthy gingiva biopsies (*n* = 20).	DEFA4 gene expression was observed to be increased (179.2-fold) in oral leukoplakia compared with healthy gingiva, suggesting that DEFA4 up-regulation may play a role in malignant transformation.

[96]	Salivary glands tumors	To determine new genes associated with proliferation in salivary glands tumors. Gene expression of (DEFA1, 2, 3, 4) was assessed in different types of salivary glands tumors and inflamed salivary gland tissues compared with healthy tissues (*n* = 10 each).	DEFA4 showed up-regulation gene expression in all salivary gland tumors subsets as well as inflamed salivary gland tissues, but a significant down-regulation in pleomorphic adenomas (benign mixed tumor).

[99]	Philadelphia-negative chronic myeloproliferative neoplasms (MPNs)	The transcriptional profiling study was conducted to investigate gene expression of interferon-associated genes using blood samples from patients with different phenotypes of MPNs: ET (*n* = 19), PV (*n* = 41), and PMF (*n* = 9) compared with controls (*n* = 21).	DEFA4 was one of the top ten up-regulated genes in patients with PMF compared with controls.

[100]	Philadelphia-negative chronic myeloproliferative neoplasms (MPNs)	To identify the gene signature that may indicate the transitional stage towards myelofibrosis and/or aggressive clinical phenotype, blood transcriptional profiling and hierarchical cluster analysis have been performed in patients with ET (*n* = 19), PV (*n* = 41), and PMF (*n* = 9), compared with controls (*n* = 21).	-DEFA4 was one of the top 20 up-regulated genes that were highly significantly deregulated only in PMF. DEFA4 was one of the five distinct gene signatures that can indicate the transition of the diseases from ET and PV towards myelofibrosis or aggressive phenotype.

## References

[1] Anaya J.-M., Shoenfeld Y., Rojas-Villarraga A., Levy R. A., Cervera R. (2013). *Autoimmunity: From Bench to Bedside*.

[2] Hazlett L., Wu M. (2011). Defensins in innate immunity. *Cell and Tissue Research*.

[3] Liu L., Zhao C., Heng H. H., Ganz T. (1997). The human *β*-defensin-1 and *α*-defensins are encoded by adjacent genes: two peptide families with differing disulfide topology share a common ancestry. *Genomics*.

[4] Palfree R. G., Sadro L. C., Solomon S. (1993). The gene encoding the human corticostatin HP-4 precursor contains a recent 86-base duplication and is located on chromosome 8. *Molecular Endocrinology*.

[5] Prasad S. V., Fiedoruk K., Daniluk T., Piktel E., Bucki R. (2019). Expression and function of host defense peptides at inflammation sites. *International Journal of Molecular Sciences*.

[6] Nusbaum C., Mikkelsen T. S., Zody M. C. (2006). DNA sequence and analysis of human chromosome 8. *Nature*.

[7] Lisitsyn N. A., Bukurova Y. A., Nikitina I. G., Krasnov G. S., Sykulev Y., Beresten S. F. (2012). Enteric alpha defensins in norm and pathology. *Annals of Clinical Microbiology and Antimicrobials*.

[8] Ganz T., Selsted M. E., Szklarek D. (1985). Defensins. natural peptide antibiotics of human neutrophils. *Journal of Clinical Investigation*.

[9] Singh A., Bateman A., Zhu Q. Z., Shimasaki S., Esch F., Solomon S. (1988). Structure of a novel human granulocyte peptide with anti-ACTH activity. *Biochemical and Biophysical Research Communications*.

[10] Agerberth B., Charo J., Werr J. (2000). The human antimicrobial and chemotactic peptides LL-37 and *α*-defensins are expressed by specific lymphocyte and monocyte populations. *Blood*.

[11] Hancock R. E. W., Haney E. F., Gill E. E. (2016). The immunology of host defence peptides: beyond antimicrobial activity. *Nature Reviews Immunology*.

[12] Rodríguez García M., Oliva H., Climent N., Garcia F., Gatell J. M., Gallart T. (2007). Human immature monocyte derived dendritic cells produce and secrete *α* defensins 1–3. *Journal of Leukocyte Biology*.

[13] Wilde C. G., Griffith J. E., Marra M. N., Snable J. L., Scott R. W. (1989). Purification and characterization of human neutrophil peptide 4, a novel member of the defensin family. *Journal of Biological Chemistry*.

[14] Ericksen B., Wu Z., Lu W., Lehrer R. I. (2005). Antibacterial activity and specificity of the six human *α*-defensins. *Antimicrobial Agents and Chemotherapy*.

[15] Wu Z., Cocchi F., Gentles D. (2005). Human neutrophil *α* defensin 4 inhibits HIV 1 infection in vitro. *FEBS Letters*.

[16] Szyk A., Wu Z., Tucker K., Yang D., Lu W., Lubkowski J. (2006). Crystal structures of human *α* defensins HNP4, HD5, and HD6. *Protein Science: A Publication of the Protein Society*.

[17] Chaly Y. V., Paleolog E. M., Kolesnikova T. S., Tikhonov I. I., Petratchenko E. V., Voitenok N. N. (2000). Neutrophil alpha-defensin human neutrophil peptide modulates cytokine production in human monocytes and adhesion molecule expression in endothelial cells. *European Cytokine Network*.

[18] Miles K., Clarke D. J., Lu W. (2009). Dying and necrotic neutrophils are anti-inflammatory secondary to the release of *α*-defensins. *The Journal of Immunology*.

[19] Brook M., Tomlinson G. H., Miles K. (2016). Neutrophil-derived alpha defensins control inflammation by inhibiting macrophage mRNA translation. *Proceedings of the National Academy of Sciences of the United States of America*.

[20] Ahn J. K., Huang B., Bae E. K. (2013). The role of *α*-defensin-1 and related signal transduction mechanisms in the production of IL-6, IL-8 and MMPs in rheumatoid fibroblast-like synoviocytes. *Rheumatology*.

[21] Gursoy U. K., Kononen E., Luukkonen N., Uitto V. J. (2013). Human neutrophil defensins and their effect on epithelial cells. *Journal of Periodontology*.

[22] Aarbiou J., Verhoosel R. M., Van Wetering S. (2004). Neutrophil defensins enhance lung epithelial wound closure and mucin gene expression in vitro. *American Journal of Respiratory Cell and Molecular Biology*.

[23] Oono T., Shirafuji Y., Huh W. K., Akiyama H., Iwatsuki K. (2002). Effects of human neutrophil peptide-1 on the expression of interstitial collagenase and type I collagen in human dermal fibroblasts. *Archives of Dermatological Research*.

[24] Hoppe T., Kraus D., Novak N. (2016). Oral pathogens change proliferation properties of oral tumor cells by affecting gene expression of human defensins. *Tumour biology: The Journal of the International Society for Oncodevelopmental Biology and Medicine*.

[25] Gaspar D., Freire J. M., Pacheco T. R., Barata J. T., Castanho M. A. R. B. (2015). Apoptotic human neutrophil peptide-1 anti-tumor activity revealed by cellular biomechanics. *Biochimica et Biophysica Acta*.

[26] Economopoulou M., Bdeir K., Cines D. B. (2005). Inhibition of pathologic retinal neovascularization by *α*-defensins. *Blood*.

[27] Xu N., Wang Y. S., Pan W. B. (2008). Human *α*-defensin-1 inhibits growth of human lung adenocarcinoma xenograft in nude mice. *Molecular Cancer Therapeutics*.

[28] Chavakis T., Cines D. B., Rhee J. S. (2004). Regulation of neovascularization by human neutrophil peptides (*α* defensins): a link between inflammation and angiogenesis. *The FASEB Journal: Official Publication of the Federation of American Societies for Experimental Biology*.

[29] Harwig S., Park A., Lehrer R. (1992). Characterization of defensin precursors in mature human neutrophils. *Blood*.

[30] Hu H., Di B., Tolbert W. D. (2019). Systematic mutational analysis of human neutrophil *α*-defensin HNP4. *Biochimica et Biophysica Acta (BBA)—Biomembranes*.

[31] Ehmann D., Wendler J., Koeninger L. (2019). Paneth cell *α*-defensins HD-5 and HD-6 display differential degradation into active antimicrobial fragments. *Proceedings of the National Academy of Sciences of the United States of America*.

[32] Böffert R., Businger R., PreiB H. (2020). The human *α*-defensin-derived peptide HD5 (1–9) inhibits cellular attachment and entry of human cytomegalovirus. *Antiviral Research*.

[33] Ehmann D., Koeninger L., Wendler J. (2020). Fragmentation of human neutrophil *α*-defensin 4 to combat multidrug resistant bacteria. *Frontiers in Microbiology*.

[34] Gabay J. E., Scott R. W., Campanelli D. (1989). Antibiotic proteins of human polymorphonuclear leukocytes. *Proceedings of the National Academy of Sciences of the United States of America*.

[35] Wu Z., Ericksen B., Tucker K., Lubkowski J., Lu W. (2004). Synthesis and characterization of human *α* defensins 4–6. *The Journal of Peptide Research: Official Journal of the American Peptide Society*.

[36] Liu T., Wu H., Cao D. (2015). Oviduct-specific expression of human neutrophil defensin 4 in lentivirally generated transgenic chickens. *PLoS One*.

[37] Ramesh P., Veerappapillai S., Karuppasamy R. (2021). Gene expression profiling of corona virus microarray datasets to identify crucial targets in COVID-19 patients. *Gene Reports*.

[38] Hemmat N., Derakhshani A., Bannazadeh Baghi H., Silvestris N., Baradaran B., De Summa S. (2020). Neutrophils, crucial, or harmful immune cells involved in coronavirus infection: a bioinformatics study. *Frontiers in Genetics*.

[39] Tsai C.-J., Ma B., Nussinov R. (2009). Protein–protein interaction networks: how can a hub protein bind so many different partners?. *Trends in Biochemical Sciences*.

[40] Buturovic L., Hong Z., Tang B. (2022). A 6-mRNA host response classifier in whole blood predicts outcomes in COVID-19 and other acute viral infections. *Scientific Reports*.

[41] The National Center for Biotechnology Information (2020). *DEFA4 Defensin Alpha 4 [Homo sapiens (Human)]*.

[42] Cunliffe R. N., Mahida Y. R. (2004). Expression and regulation of antimicrobial peptides in the gastrointestinal tract. *Journal of Leukocyte Biology*.

[43] Ensembl (2021). *Gene: DEFA4 ENSG00000164821*.

[44] Genome Browser Gateway (2020). *Gene Expression in 54 Tissues from GTEx RNA-Seq of 17382 Samples, 948 Donors (V8, Aug 2019) (DEFA4)*.

[45] (2021). *The Universal Protein Resource (UniProt), ProtKB—P12838 (DEF4_HUMAN)*.

[46] (2021). *UniProt, U.P.K. B. - P12838 (DEF4_HUMAN)*.

[47] Allen M. J., Sharma S. (2019). Physiology, adrenocorticotropic hormone (ACTH). *StatPearls*.

[48] Centers for Disease Control and Prevention (2019). *Antibiotic Resistance Threats in the United States*.

[49] Tacconelli E., Carrara E., Savoldi A. (2018). Discovery, research, and development of new antibiotics: the WHO priority list of antibiotic-resistant bacteria and tuberculosis. *The Lancet Infectious Diseases*.

[50] Moss J. A. (2013). HIV/AIDS review. *Radiologic Technology*.

[51] Furci L., Sironi F., Tolazzi M., Vassena L., Lusso P. (2007). Alpha-defensins block the early steps of HIV-1 infection: interference with the binding of gp120 to CD4. *Blood*.

[52] Wang W., Owen S. M., Rudolph D. L. (2004). Activity of *α*-and *θ*-defensins against primary isolates of HIV-1. *The Journal of Immunology*.

[53] Zhou Q., Ding W., Jiang L. (2016). Comparative transcriptome analysis of peripheral blood mononuclear cells in hepatitis B-related acute-on-chronic liver failure. *Scientific Reports*.

[54] Qiu S., He P., Fang X. (2018). Significant transcriptome and cytokine changes in hepatitis B vaccine non-responders revealed by genome-wide comparative analysis. *Human Vaccines & Immunotherapeutics*.

[55] Klotman M. E., Chang T. L. (2006). Defensins in innate antiviral immunity. *Nature Reviews Immunology*.

[56] Muffak-Granero K., Bueno P., Olmedo C. (2008). Study of gene expression profile in liver transplant recipients with hepatitis C virus. *Transplantation Proceedings*.

[57] Ramdasi A. Y., Arankalle V. A. (2020). The expression patterns of immune response genes in the Peripheral Blood Mononuclear cells of pregnant women presenting with subclinical or clinical HEV infection are different and trimester-dependent: a whole transcriptome analysis. *PLoS One*.

[58] Knight A. K., Dunlop A. L., Kilaru V. (2018). Characterization of gene expression changes over healthy term pregnancies. *PLoS One*.

[59] Overmyer K. A., Shishkova E., Miller I. J. (2021). Large-scale multi-omic analysis of COVID-19 severity. *Cell systems*.

[60] Cavalcante-Silva L. H. A., Carvalho D. C. M., Lima E. D. A. (2020). Neutrophils and COVID-19: the road so far. *International Immunopharmacology*.

[61] Zuo Y., Yalavarthi S., Shi H. (2020). Neutrophil extracellular traps in COVID-19. *JCI insight*.

[62] Abdeen S., Bdeir K., Abu-Fanne R. (2021). Alpha defensins: risk factor for thrombosis in COVID 19 infection. *British Journal of Haematology*.

[63] Wilk A. J., Lee M. J., Wei B. (2021). Multi-omic profiling reveals widespread dysregulation of innate immunity and hematopoiesis in COVID-19. *Journal of Experimental Medicine*.

[64] Group R. C. (2021). Dexamethasone in hospitalized patients with Covid-19. *New England Journal of Medicine*.

[65] Cain D. W., Cidlowski J. A. (2017). Immune regulation by glucocorticoids. *Nature Reviews Immunology*.

[66] Alzahrani A. S., Mukhtar N., Aljomaiah A. (2021). The impact of COVID-19 viral infection on the hypothalamic-pituitary-adrenal axis. *Endocrine Practice: Official Journal of the American College of Endocrinology and the American Association of Clinical Endocrinologists*.

[67] Bigler J., Boedigheimer M., Schofield J. P. R. (2017). A severe asthma disease signature from gene expression profiling of peripheral blood from U-BIOPRED cohorts. *American Journal of Respiratory and Critical Care Medicine*.

[68] Su M. W., Lin W. C., Tsai C. H. (2018). Childhood asthma clusters reveal neutrophil predominant phenotype with distinct gene expression. *Allergy*.

[69] Yang I. V., Luna L. G., Cotter J. (2012). The peripheral blood transcriptome identifies the presence and extent of disease in idiopathic pulmonary fibrosis. *PLoS One*.

[70] Molyneaux P. L., Willis-Owen S. A. G., Cox M. J. (2017). Host-microbial interactions in idiopathic pulmonary fibrosis. *American Journal of Respiratory and Critical Care Medicine*.

[71] Porto B. N., Stein R. T. (2016). Neutrophil extracellular traps in pulmonary diseases: too much of a good thing?. *Frontiers in Immunology*.

[72] Saffarzadeh M., Juenemann C., Queisser M. A. (2012). Neutrophil extracellular traps directly induce epithelial and endothelial cell death: a predominant role of histones. *PLoS One*.

[73] Jourdain M. L., Pierrard L., Kanagaratnam L. (2018). Antimicrobial peptide gene expression in periodontitis patients: a pilot study. *Journal of Clinical Periodontology*.

[74] Villanueva E., Yalavarthi S., Berthier C. C. (2011). Netting neutrophils induce endothelial damage, infiltrate tissues, and expose immunostimulatory molecules in systemic lupus erythematosus. *The Journal of Immunology*.

[75] O’Hanlon T. P., Rider L. G., Gan L. (2011). Gene expression profiles from discordant monozygotic twins suggest that molecular pathways are shared among multiple systemic autoimmune diseases. *Arthritis Research and Therapy*.

[76] Craddock R. M., Huang J. T., Jackson E. (2008). Increased *α*-defensins as a blood marker for schizophrenia susceptibility. *Molecular & Cellular Proteomics: MCP*.

[77] Gardiner E. J., Cairns M. J., Liu B. (2013). Gene expression analysis reveals schizophrenia-associated dysregulation of immune pathways in peripheral blood mononuclear cells. *Journal of Psychiatric Research*.

[78] Iavarone F., Melis M., Platania G. (2014). Characterization of salivary proteins of schizophrenic and bipolar disorder patients by top-down proteomics. *Journal of Proteomics*.

[79] Köhler C. A., Freitas T. H., Maes M. (2017). Peripheral cytokine and chemokine alterations in depression: a meta analysis of 82 studies. *Acta Psychiatrica Scandinavica*.

[80] Hori H., Yoshida F., Itoh M. (2020). Proinflammatory status-stratified blood transcriptome profiling of civilian women with PTSD. *Psychoneuroendocrinology*.

[81] Cohen D., Pilozzi A., Huang X. (2020). Network medicine approach for analysis of Alzheimer’s disease gene expression data. *International Journal of Molecular Sciences*.

[82] Infante J., Prieto C., Sierra M. (2015). Identification of candidate genes for Parkinson’s disease through blood transcriptome analysis in LRRK2-G2019S carriers, idiopathic cases, and controls. *Neurobiology of Aging*.

[83] Fan Y., Li J., Yang Q. (2019). Dysregulated long non-coding RNAs in Parkinson’s disease contribute to the apoptosis of human neuroblastoma cells. *Frontiers in Neuroscience*.

[84] Benedicto C.-F., Prieto C., Sainz J. (2019). Altered gene expression in antipsychotic-induced weight gain. *NPJ Schizophrenia*.

[85] Bradburn S., Sarginson J., Murgatroyd C. A. (2017). Association of peripheral interleukin-6 with global cognitive decline in non-demented adults: a meta-analysis of prospective studies. *Frontiers in Aging Neuroscience*.

[86] Lyra e Silva N. M., Rafaella A. G., Tharick A. P. (2021). Pro-inflammatory interleukin-6 signaling links cognitive impairments and peripheral metabolic alterations in Alzheimer’s disease. *Translational Psychiatry*.

[87] Al-Hakeim H. K., Al-Rammahi D. A., Al-Dujaili A. H. (2015). IL-6, IL-18, sIL-2R, and TNF*α* proinflammatory markers in depression and schizophrenia patients who are free of overt inflammation. *Journal of Affective Disorders*.

[88] Banks W. A., Kastin A. J., Gutierrez E. G. (1994). Penetration of interleukin-6 across the murine blood-brain barrier. *Neuroscience Letters*.

[89] Piasecka M., Papakokkinou E., Valassi E. (2020). Psychiatric and neurocognitive consequences of endogenous hypercortisolism. *Journal of Internal Medicine*.

[90] Gatti G., Masera R. G., Bateman A. (1992). Effects of CRH, acth and the corticostatin HP-4 on the spontaneous NK cell activity and susceptibility to endogenous modifiers. *Archives of Gerontology and Geriatrics*.

[91] Myhr K. M., Mellgren S. I. (2009). Corticosteroids in the treatment of multiple sclerosis. *Acta Neurologica Scandinavica—Supplement*.

[92] Irizar H., Munoz-Culla M., Sepulveda L. (2014). Transcriptomic profile reveals gender-specific molecular mechanisms driving multiple sclerosis progression. *PLoS One*.

[93] De Andres C., Garcia M. I., Goicoechea H. (2018). Genes differentially expressed by methylprednisolone in vivo in CD4 T lymphocytes from multiple sclerosis patients: potential biomarkers. *The Pharmacogenomics Journal*.

[94] Melief J., Orre M., Bossers K. (2019). Transcriptome analysis of normal-appearing white matter reveals cortisol-and disease-associated gene expression profiles in multiple sclerosis. *Acta neuropathologica communications*.

[95] Xu L., Geman D., Winslow R. L. (2007). Large-scale integration of cancer microarray data identifies a robust common cancer signature. *BMC Bioinformatics*.

[96] Winter J., Pantelis A., Kraus D. (2012). Human *α*-defensin (DEFA) gene expression helps to characterise benign and malignant salivary gland tumours. *BMC Cancer*.

[97] Wenghoefer M., Pantelis A., Najafi T. (2010). Gene expression of oncogenes, antimicrobial peptides, and cytokines in the development of oral leukoplakia. *Oral Surgery, Oral Medicine, Oral Pathology, Oral Radiology & Endodontics*.

[98] Mohammed F., Fairozekhan A. T. (2017). *Oral Leukoplakia*.

[99] Skov V., Larsen T. S., Thomassen M. (2011). Whole blood transcriptional profiling of interferon inducible genes identifies highly upregulated IFI27 in primary myelofibrosis. *European Journal of Haematology*.

[100] Hasselbalch H. C., Skov V., Stauffer Larsen T. (2014). Transcriptional profiling of whole blood identifies a unique 5-gene signature for myelofibrosis and imminent myelofibrosis transformation. *PLoS One*.

